# Draft Genome of *Scalindua rubra*, Obtained from the Interface Above the Discovery Deep Brine in the Red Sea, Sheds Light on Potential Salt Adaptation Strategies in Anammox Bacteria

**DOI:** 10.1007/s00248-017-0929-7

**Published:** 2017-01-10

**Authors:** Daan R. Speth, Ilias Lagkouvardos, Yong Wang, Pei-Yuan Qian, Bas E. Dutilh, Mike S. M. Jetten

**Affiliations:** 1 Department of Microbiology, Institute for Water and Wetland Research, Radboud University, Nijmegen, The Netherlands; 2 ZIEL Institute for Food and Health, Technische Universität München, Freising, Germany; 3 Institute of Deep-Sea Science and Engineering, Chinese Academy of Sciences, Sanya, China; 4 Division of Life Science, Hong Kong University of Science and Technology, Clear Water Bay, Hong Kong; 5 Theoretical Biology and Bioinformatics, Utrecht University, Utrecht, The Netherlands; 6 Centre for Molecular and Biomolecular Informatics, Radboud University Medical Centre, Nijmegen, The Netherlands; 7 Instituto de Biologia, Universidade Federal do Rio de Janeiro, Rio de Janeiro, Brazil; 8 Department of Biotechnology, Delft University of Technology, Delft, The Netherlands; 9 Division for Geological and Planetary Sciences, California Institute of Technology, Pasadena, CA USA

**Keywords:** Scalindua, Anammox, Red Sea, Genome binning, Metagenomics, Salt adaptation

## Abstract

Several recent studies have indicated that members of the phylum *Planctomycetes* are abundantly present at the brine-seawater interface (BSI) above multiple brine pools in the Red Sea. *Planctomycetes* include bacteria capable of anaerobic ammonium oxidation (anammox). Here, we investigated the possibility of anammox at BSI sites using metagenomic shotgun sequencing of DNA obtained from the BSI above the Discovery Deep brine pool. Analysis of sequencing reads matching the 16S rRNA and *hzsA* genes confirmed presence of anammox bacteria of the genus *Scalindua*. Phylogenetic analysis of the 16S rRNA gene indicated that this *Scalindua* sp. belongs to a distinct group, separate from the anammox bacteria in the seawater column, that contains mostly sequences retrieved from high-salt environments. Using coverage- and composition-based binning, we extracted and assembled the draft genome of the dominant anammox bacterium. Comparative genomic analysis indicated that this *Scalindua* species uses compatible solutes for osmoadaptation, in contrast to other marine anammox bacteria that likely use a salt-in strategy. We propose the name *Candidatus* Scalindua rubra for this novel species, alluding to its discovery in the Red Sea.

**Electronic supplementary material:**

The online version of this article (doi:10.1007/s00248-017-0929-7) contains supplementary material, which is available to authorized users.

Over 25 brine pools have been discovered along the rift through the middle of the Red Sea. These brine pools are characterized by anoxic, salty water, and in some cases geothermal activity [1]. The high salinity of the brine pools prevents mixing with the overlying seawater creating a brine-seawater interface (BSI) featuring steep salt and, in the case of hot brines, temperature gradients. Several studies using 16S rRNA gene amplicon community profiling and shotgun metagenomics have recently revealed the abundant presence of *Planctomycetes* (5–35%) in the BSI above the Discovery Deep, Atlantis II Deep, and Kebrit Deep brine pools [2–4]. As these are low-oxygen environments, detection of *Planctomycetes* likely indicates the presence of anammox bacteria. Furthermore, recent studies have shown the presence of ammonia-oxidizing *Archaea* and nitrite-oxidizing *Bacteria* in the Atlantis II Deep BSI, indicating an active nitrogen cycle in these systems [5, 6]. To further investigate the presence and nature of anammox bacteria in the Red Sea BSI, we employed genome-resolved shotgun metagenomics of the BSI above the Discovery Deep, where 16S rRNA gene amplicon community profiling indicated that *Planctomycetes* were more abundant than in other brine pools [2].

Total microbial community DNA (sample DIS-BWI, see [2] for sampling and DNA extraction) was prepared for IonTorrent sequencing as previously described [7]. The resulting library was used for two sequencing runs, resulting in a total of 10.1 million single-end reads. Reads were trimmed on quality (quality limit = 0.05) and length (>100 bp) using CLCgenomics workbench (v8.0.3, CLCbio, Arhus, Denmark). The presence of anammox was investigated by reconstructing full-length sequences of the 16S rRNA and hydrazine synthase alpha (*hzsA*) genes by mapping and assembly using the CLCgenomics workbench, as described previously [8]. Two 16S rRNA genes matching the *Scalindua* clade, with coverage 52× and 14×, and two *hzsA* sequences, with coverage 37× and 16×, could be reconstructed. The 16S sequence obtained from the former, dominant Scalindua species (5.7% of all 16S rRNA gene reads in our dataset), hereafter referred to as *Candidatus* Scalindua rubra, is only 94% identical to *Candidatus* Scalindua brodae and clusters with sequences obtained from the Atlantis II Deep BSI, the brine adjacent to the Discovery Deep [4] (Fig. 1a). The latter, low abundant *Scalindua* species (1.3% of all rRNA gene 16S reads) clusters with sequences from the Arabian Sea oxygen minimum zone and other sequences obtained from the Atlantis II deep (Fig. 1a). Previously sequenced *Ca*. S. brodae [9] and *Candidatus* Scalindua profunda [10] formed a third cluster that also includes most sequences obtained from the Eastern Tropical South Pacific oxygen minimum zone (Fig. 1a). Phylogenetic analysis of the *hzsA* genes corroborates that *Ca*. S. rubra is distant from *Ca*. S. profunda and *Ca*. S. brodae (Fig. 1b). Interestingly, the partial sequences 16S rRNA and *hzsA* sequences obtained by Borin et al. [11] from the chemocline above Bannock brine in the Mediterranean, cluster with *Ca*. S. brodae and *Ca*. S. profunda, rather than with the sequences obtained from the Atlantis II Deep and Discovery Deep BSI.

**Fig. 1:**
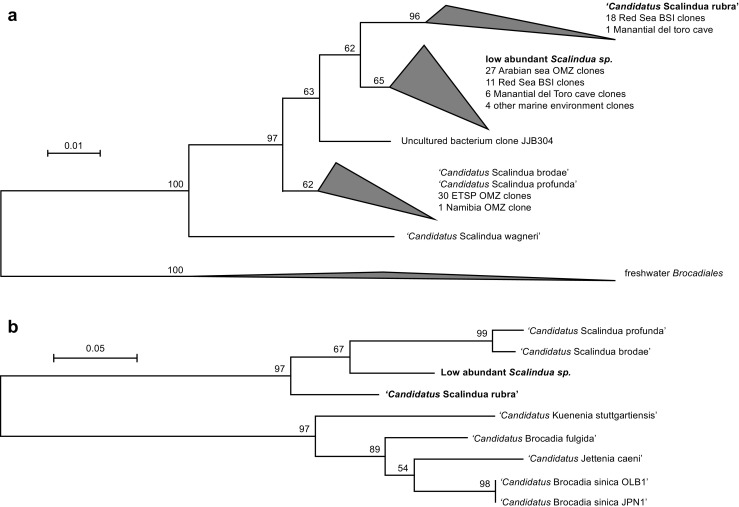
Maximum likelihood trees of anammox 16S rRNA and *hzsA* genes. **a** Maximum likelihood tree of 109 near full-length *Brocadiales* 16S rRNA genes matching >90% of the length the *Ca*. S. rubra sequence, originating from enrichment cultures, draft genomes, and clone libraries of marine environments. **b** Maximum likelihood tree of all available full-length *hzsA* gene sequences obtained from draft genomes. Sequences obtained in this study are indicated in *bold*. Trees were constructed using MEGA5 [36], bootstrapped with 1000 replicates, and visualized using the interactive tree of life (iTOL) v3 webserver [37]. Wedge height was scaled proportional to number of sequences. *OMZ* oxygen minimum zone, *BSI* brine-seawater interface, *ETSP* Eastern Tropical South Pacific

To obtain a draft genome of *Ca*. S. rubra, we assembled the metagenome de novo using the CLCgenomics workbench with word size 31 and bubble size 5000. Contigs were assigned to *Ca*. S. rubra using emergent self-organizing maps [12, 13], coverage, and GC content [14]. Scripts used for binning are available at www.github.com/dspeth. The resulting 1020 *Ca*. S. rubra contigs were used for iterative reassembly using SPAdes (version 3.5.0) [15] and Bowtie2 [16], resulting in 443 contigs assigned to *Ca*. S. rubra (Table 1). Contigs were error corrected to account for persistent IonTorrent-specific errors as described previously [7] and annotated using Prokka (version 1.10) [17] using a custom database containing the six *Brocadiales* draft genomes in Genbank [7, 9, 18–21]. Coverage of the contigs representing the low-abundance *Scalindua* species was too low (∼15×) to enable extraction of a good quality draft genome of this organism.

**Table 1:** Metrics of the available *Scalindua* spp. draft genomes

Species	Genome size (Mbp)	GC content	Completeness (%)	Contamination (%)	# of contigs	Reference
*Candidatus* Scalindua profunda	5.14	39.1	95	3	1580	[10]
*Candidatus* Scalindua brodae	4.08	39.6	92	2.3	282	[9]
*Candidatus* Scalindua rubra	5.19	37.3	92	5.1	443	This study

Completeness and contamination of the *Scalindua* spp. draft genomes were estimated using checkM [35]

The *Ca*. S. rubra draft genome encoded the genes required for hydrazine metabolism, hydrazine synthase [22] (SCARUB_01028–SCARUB_01030), and hydrazine dehydrogenase [23] (SCARUB_00654). The genes encoding hydrazine synthase subunits B and C are not fused in *Ca*. S. rubra, suggesting that the fusion of these genes in *Ca*. S. profunda and *Ca*. S. brodae is a recent event. Like the other *Scalindua* species, *Ca*. S. rubra encodes a heme-*cd*_*1*_ type nitrite reductase (nirS) (SCARUB_03231). In contrast to *Ca*. S. profunda, neither *Ca*. S. brodae nor *Ca*. S. rubra encode a cyanase. Another interesting feature in the *Ca*. S. rubra genome is the apparent capability to synthesize gas vesicles, as 11 gas vesicle synthesis proteins are present. Although gas vesicles are often regulated by light intensity, gas vesicle formation is induced by high salinity in halophilic *Archaeon Haloferax mediterranei* [24]. It is possible that *Ca*. S. rubra uses gas vesicles to stabilize its position within the BSI and prevent osmotic and/or heat shock as a result of the steep gradients in the BSI. The cellular location of gas vesicles in the already complicated cell architecture of an anammox bacterium is an interesting topic for further investigation.

To assess further adaptations to life in the BSI we searched the draft genome of *Ca*. S. rubra for mechanisms of osmoadaptation. Based on the recent work of Ngugi and colleagues [5], we used protein isoelectric point (IEP) distributions as indicator for a charged cytoplasm resulting from a “salt-in” osmoadaptation strategy [25]. We calculated the IEP of all predicted proteins in the eight available genomes of anammox bacteria using the “iep” script from the EMBOSS package (v6.5.7) [26]. Surprisingly, the median protein IEP of *Ca*. S. rubra is more basic than the median protein IEP of *Ca*. S. brodae and *Ca*. S. profunda and comparable to that of the freshwater species (Fig. 2). The acid-shifted distribution of protein IEP indicates that both previously sequenced *Scalindua* species have adapted to seawater salinity using a “salt-in” strategy, adding acidic residues to prevent protein denaturation in high-ion concentrations [27]. The observations that, in contrast to freshwater species, salt in the growth medium was required to enrich *Ca*. S. profunda and that 90% of dry weight of this organism consisted of salt further support this interpretation [28, 29]. As expected, the acid shift is more pronounced if only cytoplasmic proteins are considered, and absent from membrane proteins (Supplemental Figure S1). In contrast, the more basic IEP of *Ca*. S. rubra proteins suggests that it relies on compatible solutes to cope with the salinity at the BSI. Synthesis of compatible solutes is energetically more costly than coping with salinity using a salt-in strategy [30]. Although some halophiles use a salt-in strategy at higher salinity than observed at the Discovery Deep BSI [31, 32], it is possible that *Ca*. S. rubra uses compatible solutes to adapt to the range of salt concentrations resulting from the steep salt gradient in the Discovery Deep BSI [2]. In line with this hypothesis, the recently published genomes of ammonia-oxidizing *Archaea* and nitrite-oxidizing *Bacteria* from the BSI above the Atlantis II Deep, which is adjacent to the Discovery Deep, also indicate that these organisms employ compatible solutes [5, 6].

**Fig. 2:**
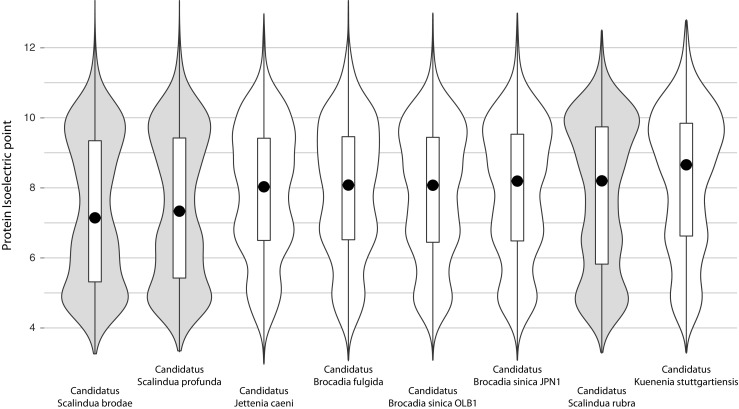
Protein isoelectric point distribution in eight genomes of anammox bacteria. Violin plots indicating the isoelectric point distribution of total protein set of all eight available anammox genomes, ordered from lowest to highest median value. Box plots (*white bars*) indicate 50% of the values around the median, indicated by a *black circle*. The three available genomes of *Scalindua* sp. are indicated by *gray shading*

We searched the *Ca*. S. rubra draft genome for proteins required for biosynthesis and transport of common compatible solutes. Many organisms use the amino acids glutamate, glutamine, or proline as compatible solutes [33]. All anammox bacteria can synthesize these amino acids, and thus, it is possible that *Ca*. S. rubra utilizes any or all three of these amino acids. This could also provide an explanation for the adaptation of freshwater anammox species *Ca*. K. stuttgartiensis to marine salt concentrations [34]. None of the *Scalindua* species is capable of synthesizing amino acid-derived compatible solutes glycine-betaine or (hydroxy)ectoine, but all three encode a glycine-betaine transporter. Furthermore, none of the *Scalindua* genomes encode the potential for biosynthesis of glycerate-derived compatible solutes or mannitol or sorbitol [33]. Conclusive evidence on the presence, and nature, of compatible solutes in *Ca*. S. rubra will require biomass for experimental verification of the amino acid content.

In conclusion, we have presented the draft genome of a moderately halophilic anammox bacterium, *Ca*. S. rubra. Our analysis of the adaptations to salt stress in this genome has shed new light on previous results of salt adaptation in anammox bacteria.

## Electronic Supplementary Material

Below is the link to the electronic supplementary material.

ESM 1 (PDF 779 kb)
