# Supplementary material for: Draft Genome of Scalindua rubra, Obtained from the Interface Above the Discovery Deep Brine in the Red Sea, Sheds Light on Potential Salt Adaptation Strategies in Anammox Bacteria
Source: Microb Ecol. 2017 Jan 10;74(1):1–5. doi: 10.1007/s00248-017-0929-7 (PMC5486813; doi:10.1007/s00248-017-0929-7)
Supplement: Supplementary file 1 — (PDF 779 kb) [file 248_2017_929_MOESM1_ESM.pdf]

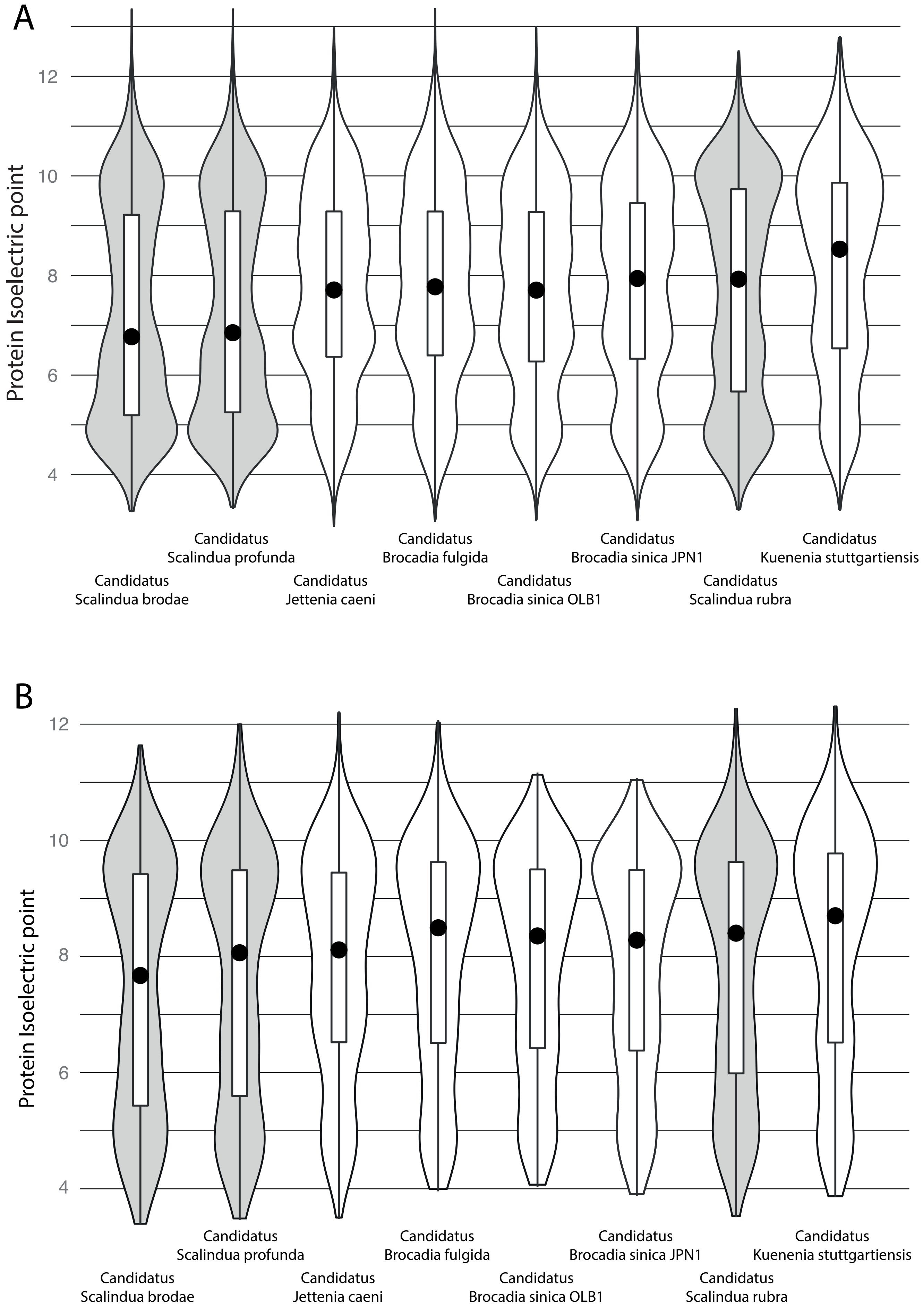
A
Protein Isoelectric point
4
6
8
10
12
Candidatus
Scalindua brodae
Candidatus
Scalindua profunda
Candidatus
Jettenia caeni
Candidatus
Brocadia fulgida
Candidatus
Brocadia sinica OLB1
Candidatus
Brocadia sinica JPN1
Candidatus
Scalindua rubra
Candidatus
Kuenenia stuttgartiensis
B
Protein Isoelectric point
4
6
8
10
12
Candidatus
Scalindua brodae
Candidatus
Scalindua profunda
Candidatus
Jettenia caeni
Candidatus
Brocadia fulgida
Candidatus
Brocadia sinica OLB1
Candidatus
Brocadia sinica JPN1
Candidatus
Scalindua rubra
Candidatus
Kuenenia stuttgartiensis

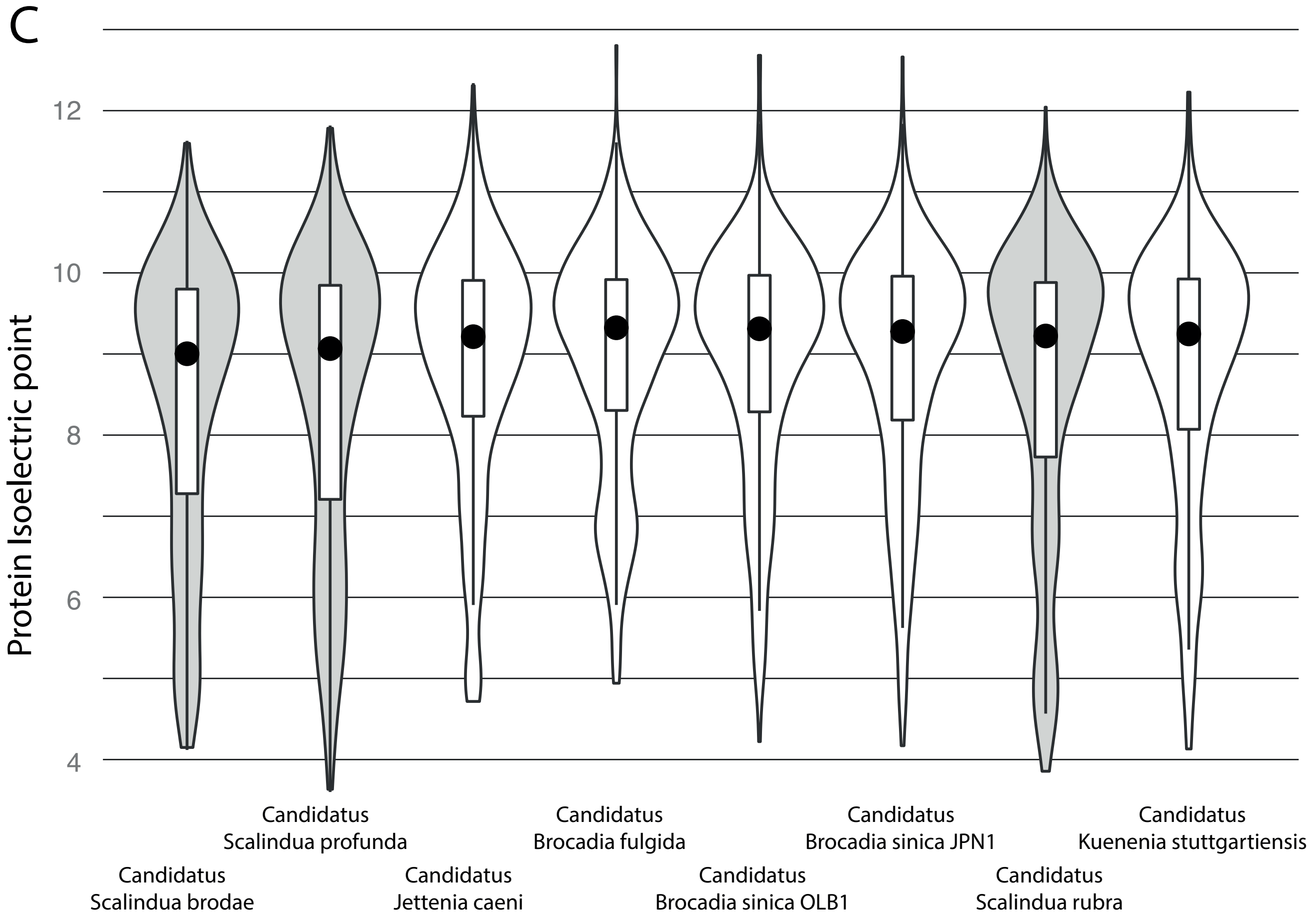

Supplemental figure S1. Protein isoelectric point distribution in eight genomes of anammox bacteria
Violin plots indicating the isoelectric point distribution of proteins in eight available anammox genomes with:
A) no predicted transmembrane helices or signal peptides
B) one predicted transmembrane helix or signal peptide
C) two or more predicted transmembrane helices
The organisms are ordered as in Figure 2, from lowest to highest median isoelectric point of the total protein set. Box plots (white bars) indicate 50% of the values around the median, indicated by a black circle. The three available genomes of Scalindua sp. are indicated by grey shading.
